# Group antenatal care models in low- and middle-income countries: a systematic evidence synthesis

**DOI:** 10.1186/s12978-018-0476-9

**Published:** 2018-03-05

**Authors:** Jigyasa Sharma, Meaghan O’Connor, R. Rima Jolivet

**Affiliations:** 1 Department of Global Health and Population, Harvard T. H. Chan School of Public Health, 677 Huntington Ave, Boston, MA 02115 USA; 2 Maternal Health Task Force, Women & Health Initiative, Harvard T.H. Chan School of Public Health, 651 Huntington Avenue, Boston, MA 02115 USA; 3 Maternal Health Task Force, Women & Health Initiative, Department of Global Health and Population, Harvard T.H. Chan School of Public Health, 651 Huntington Avenue, Boston, MA 02115 USA

**Keywords:** Antenatal care, Prenatal care, Group care during pregnancy, Systematic review, Qualitative evidence synthesis

## Abstract

In high-income countries, group antenatal care (ANC) offers an alternative to individual care and is associated with improved attendance, client satisfaction, and health outcomes for pregnant women and newborns. In low- and middle-income country (LMIC) settings, this model could be adapted to address low antenatal care uptake and improve quality. However, evidence on key attributes of a group care model for low-resource settings remains scant. We conducted a systematic review of the published literature on models of group antenatal care in LMICs to identify attributes that may increase the relevance, acceptability and effectiveness of group ANC in such settings. We systematically searched five databases and conducted hand and reference searches. We also conducted key informant interviews with researchers and program implementers who have introduced group antenatal care models in LMICs. Using a pre-defined evidence summary template, we extracted evidence on key attributes—like session content and frequency, and group composition and organization—of group care models introduced across LMIC settings. Our systematic literature review identified nine unique descriptions of group antenatal care models. We supplemented this information with evidence from 10 key informant interviews. We synthesized evidence from these 19 data sources to identify attributes of group care models for pregnant women that appeared consistently across all of them. We considered these components that are fundamental to the delivery of group antenatal care. We also identified attributes that need to be tailored to the context in which they are implemented to meet local standards for comprehensive ANC, for example, the number of sessions and the session content. We compiled these attributes to codify a composite “generic” model of group antenatal care for adaptation and implementation in LMIC settings. With this combination of standard and flexible components, group antenatal care, a service delivery alternative that has been successfully introduced and implemented in high-income country settings, can be adapted for improving provision and experiences of care for pregnant women in LMIC. Any conclusions about the benefits of this model for women, babies, and health systems in LMICs, however, must be based on robust evaluations of group antenatal care programs in those settings.

**Electronic supplementary material:**

The online version of this article (10.1186/s12978-018-0476-9) contains supplementary material, which is available to authorized users.

## Plain English summary

Antenatal care (ANC) is an important part of maternal health care, but the use of ANC services remains low in low- and middle-income countries (LMICs) and its quality poor. Evidence from high-income countries suggests that group ANC models can improve experiences of care and health outcomes for pregnant women and newborns. However, there is little systematic evidence available to guide those who want to adapt group ANC for LMICs. To fill this gap in the literature, we reviewed and gathered evidence from nine published papers and 10 interviews with researchers and programmers who are testing group ANC in LMIC settings. Using the information gathered through the review, we developed a “generic” model of group ANC for LMICs that features fixed and flexible components, making it particularly well-suited for adaptation and use in such settings.

## Background

To improve health outcomes and reduce disparities among pregnant women and newborns in low- and middle-income countries (LMICs), more must be done to increase access to quality maternal health care services for women, especially for those from vulnerable populations [1]. High quality antenatal care (ANC) optimizes both the outcomes and experiences of maternal health care for pregnant women along with outcomes for newborns. ANC is not only an opportunity for offering relevant clinical care and emotional support for pregnant women: utilization of ANC is also associated with an increased utilization of subsequent health services such as institutional delivery and postnatal care [2]. Thus, providing high quality, woman-centered ANC is especially important in LMICs that continue to bear a disproportionate burden of adverse pregnancy and newborn outcomes. In high-income countries, group ANC has emerged as an alternative service delivery model and is associated with improved attendance, satisfaction with care, and health outcomes for pregnant women and newborns, including for women from marginalized groups with perinatal outcomes that are comparable to those in some LMICs [3–9]. The predominant model of group ANC in high-income countries, CenteringPregnancy® [10], was developed in the United States to meet clinical guidelines for ANC in the US; thus, most of the evidence on use of this model comes from high-income settings.

Traditional ANC service delivery is based on one-on-one visits between a health care provider (HCP) and a pregnant woman, and focuses primarily on physical risk assessment to ensure optimal health. Within the allotted appointment time, the HCP communicates pertinent clinical and self-care (activities that individuals can perform on their own behalf) information to the woman [11]. In contrast to the traditional model of ANC delivery, group ANC is an integrated approach that incorporates physical assessment, education and skill development, and peer support. As such, it takes a broader, more holistic, woman-centered approach to ANC. Women receiving ANC in a group model benefit from both the expertise of their HCP and the knowledge, experience and support of their peers [12–14]. Thus, group ANC can be posited to fulfill key elements of a framework for woman-centered care, including the need for respect and safety; empowerment, involvement and participation of women; a collaborative, inclusive approach to the provision of health care; and an emphasis on shared information and decision making [15].

Research studies and programs currently conducting group ANC in LMIC settings are using various models that build on the strengths of other group care models, including: CenteringPregnancy®; Home-Based Life-Saving Skills (HBLSS), a program of the American College of Nurse-Midwives [10, 16]; women’s participatory action groups; shared medical appointments (SMA); and drop-in group medical appointments (DIGMA) [17–19]. Like women’s participatory action groups, group ANC can serve as a vehicle for demand generation, patient activation and community mobilization for self-care [20]. Like SMAs and DIGMAs, which are physician-centered group care models, it can increase organizational efficiencies and provider productivity [17, 19]. As such, group ANC serves as an alternative vehicle for providing woman-centered and efficient clinical care, relevant and timely pregnancy-related information, and increased emotional and social support during pregnancy.

Given its success in high-income countries, it is reasonable to hypothesize that group ANC may optimize health outcomes and experiences of care for pregnant women in LMICs as well. As a first step to exploring the effects of group ANC in a LMIC, we set out to identify critical attributes of a model designed for use in an LMIC context. We conducted a systematic review of evidence on group ANC models used in LMICs. Our objective was to explore models of group ANC used to date in LMICs, and to compile and synthesize evidence on such models to inform future studies on group ANC in LMICs.

## Methods

We conducted this systematic evidence synthesis as the first part of a larger project investigating the feasibility of group ANC in an urban setting in India [21]. The objectives of this exercise were: (1) to systematically review and catalog key attributes of group ANC models that have been implemented in LMICs, and (2) to compile and synthesize the common attributes of those models to codify a composite “generic” model for use in LMICs. Given that group ANC has only recently been introduced in LMICs and there are few published papers on the topic, we gathered evidence from two sources: (1) published literature and (2) expert consultation. We extracted the data from these sources and used the information collected to create a “generic” model of group ANC specifically for use in LMIC settings.

We used this generic model, tailoring the content and number of sessions for context, to explore the acceptability and feasibility of group ANC in urban India. The findings from that study are reported elsewhere [21].

### Literature search strategy

We systematically searched published literature in five databases (MEDLINE [Ovid], Embase, Web of Science, CINAHL, and the WHO Global Health Library) using the pre-determined strategy detailed in (Additional file 1). Searches were conducted on January 26, 2017, with date restrictions. To narrow the search results, we applied an LMIC hedge, using the World Bank country income classifications. We also searched the online bibliography available through the Centering Healthcare Institute [22] and the Maternal Health Task Force Resource Database [23] for relevant articles from LMIC settings. We hand-searched reference lists of all included studies to identify additional literature. Database search strategy terms are included as Additional File 1.

### Study selection

Each title and abstract was screened for inclusion by tw o independent reviewers (JS, MO’C) using standardized inclusion criteria: (1) the article is not an editorial, newspaper article, or other form of popular media; (2) the article describes an antenatal care intervention for pregnant women that is provided in a group setting; (3) the study takes place in a LMIC (as per World Bank classification); and (4) the article is available in English. The same criteria were applied during full-text screen. Discrepancies during title, abstract and full-text screening were resolved by discussion with a third reviewer (RJ) until consensus was reached. The number of excluded articles (including rationale for exclusion following full-text review) was recorded at each stage.

### Expert consultation

Because several studies on group ANC in LMIC are currently underway and have not yet been published, we supplemented the literature review results with data gathered via expert consultation.

We conducted semi-structured key informant interviews with researchers and programmers who are currently implementing or testing group ANC in LMICs. We identified key informants through a global research consortium on group ANC in LMIC formed at the Global Maternal Newborn Health Conference, [Mexico City, Mexico, October 18–21, 2015]. We contacted all participants in the research consortium and used referral sampling to learn from them about any others who were currently testing or implementing group ANC in a LMIC setting. All those we contacted agreed to speak with us. Ethics approval for this study was granted by the Harvard T.H. Chan School of Public Health Office of Human Research Administration. We used a set of standard guiding questions (Additional file 2) with each informant. Two researchers (MO’C and RJ) conducted the interviews.

### Data extraction and synthesis

Following the initial screening of published literature and completion of the key informant interviews, two independent reviewers (JS and MO’C) extracted data from both sources using a predefined evidence summary template. We extracted qualitative data on key attributes related to the structure and content of the models of group care (participatory groups and group ANC) for pregnant women in LMICs that we identified. We grouped attributes related to the structure and format of group models— including group composition, group leadership, dynamics and environment, and other logistical details—as well as the three basic components of the content of group care models (physical assessment, education and skill development, and peer support) from the session content of identified models. We synthesized the findings of the data extraction exercise and compiled them into a composite “generic” model of group ANC that could be adapted for use in LMIC settings. Data was analyzed by hand.

### Reporting

This review is reported following the PRISMA [24] and ENTREQ [25] statement guidelines, as applicable, to enhance transparency in reporting systematic reviews and evidence synthesis. PRISMA is an evidence-based framework for reporting in systematic reviews, which suggests minimum reporting standards for such studies. ENTREQ is a proposed standard framework for reporting the synthesis of qualitative health research.

## Results

### General overview

**Fig. 1:**
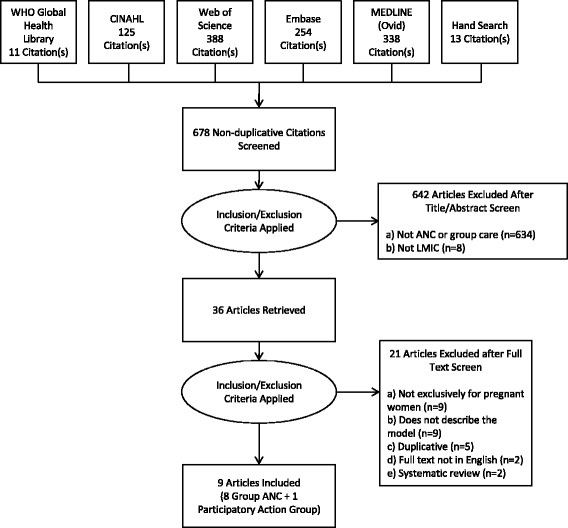
PRISMA flowchart showing study selection process

The database searches together with the hand-searches yielded 678 articles. Of these, 642 articles were excluded through title and abstract screening. Full-text was retrieved and reviewed for 36 articles. If a model was described in more than one article, we extracted information from the earliest publication and used information from the subsequent articles to supplement the data as needed. After excluding ineligible studies or studies reporting on a previously described group ANC model, nine articles [26–34], each describing a unique model of group care for pregnant women (ANC or participatory), were included for data extraction (Fig. 1). We also conducted 10 semi-structured key informant interviews. Data from some key informants supplemented data collected from their published studies reporting on the same model and vice versa [29, 30, 35]. Altogether, we describe 19 unique models of group care in this review.

This analysis synthesizes evidence from 16 countries: Bangladesh, Botswana, Brazil, Egypt, Ghana, India, Iran, Kenya, Malawi, Mexico, Nepal, Nigeria, Rwanda, Suriname, Tanzania, and Uganda. Urban and rural populations were represented, and the group care settings described included hospitals, community health centers, and clinics

Except for one article describing a participatory action group for pregnant women in India, all the articles that met our inclusion criteria and all the key informant interviews described group ANC models. A few (3/9) models described in the published literature and a majority (6/10) of models described by key informants were informed by the CenteringPregnancy® model [10] of group ANC. None of the models in the published literature and four of the 10 models described by key informants were informed by the Home Based Life Saving Skills, or HBLSS, program [16].

Table 1 displays the key attributes of group ANC models from published studies identified in the systematic review of the literature. Table 2 displays the key attributes of the group ANC models described by key informants. Panel 1 of each table summarizes the main characteristics of the research study or program from which the group model description was derived. Panel 2 of each table summarizes the specific attributes of the models of group care introduced in LMICs.

**Table 1:** Summary of evidence from published literature describing group models for antenatal care delivery in low- and middle-income countries

Study Citation	Sultana et al. (2017) [26]	Ruhl et al. (2015) [27]	Penna et al. (2008) [28]	Patil et al. (2013) [29]	Lori et al. (2016) [30]	Jafari et al. (2010) [31]	Ghani (2014) [32]	Arnold et al. (2014) [33]	Nair et al. (2015) [34]
Panel I: Study or Program Characteristics									
Group ANC or Participatory Group	Group ANC	Group ANC	Group ANC	Group ANC	Group ANC	Group ANC	Group ANC	Group ANC	Participatory Group
Country	Bangladesh	Kenya	Brazil	Malawi, Tanzania	Ghana	Iran	Egypt	Botswana	India
Setting	- Public/Nonhospital	–	–	- PublicHospital	UrbanPublicHospital	UrbanPublicNon-hospital	Urban-Hospital	- Private Hospital	Rural--
Is the model informed by an existing group format?	No	No	No	CP	No	No	CP	CP	No
Panel II: Model Attributes									
(a) Group									
How many women are in a group?	6–10 women	15–20 women	–	12 women	12 women	8–10 women	–	12 mother/father pairs	–
How are women grouped?	Gestational age	Gestational age	–	Availability and willingness to attend	Gestational age	Gestational age	Gestational age	Gestational age	Gestational age
Is the composition of the group stable?	–	–	–	Yes	Yes	–	Yes	Yes	–
(b) Leaders									
How many leaders does the group have?	2	2	–	2	–	1	–	–	–
What cadre of care providers typically leads the group?	1 Qualified HCP/1 Nurse	1 Qualified HCP/1 CHW	–	Midwives	Midwives	Midwife	–	Midwife or Women’s Health Nurse Practitioner	CHW
Is the group leadership the same throughout all sessions?	–	–	–	Yes	–	–	Yes	Yes	–
Do group leaders use a facilitative leadership style?	–	–	–	Yes	–	Yes	Yes	Yes	Yes
(c) Content									
Does the model include a clinical assessment component?	Yes	Yes	Yes	Yes	Yes	Yes	Yes	Yes	No
Does the model include an education or skill-building component?	Yes	Yes	Yes	Yes	Yes	Yes	Yes	Yes	Yes
Does the model include a support group or counselling component?	Yes	Yes	Yes	Yes	Yes	Yes	Yes	Yes	Yes
Does the health assessment occur within the group space?	–	–	Yes	Yes	Yes	No	Yes	Yes	No
Do the women participate in self-care activities?	–	–	Yes	Yes	–	Yes	Yes	Yes	No
Are sessions guided by an overall plan or outline?	Yes	–	–	Yes	Yes	Yes	Yes	Yes	–
Do sessions follow the core content but allow flexibility to tailor the session to the group’s needs?	–	–	–	Yes	Yes	–	Yes	Yes	–
Does the model include a postnatal care component?	No	Yes (4 PNC)	–	No	No	No	–	Yes (1 PNC)	–
(d) Dynamics and Environment									
Does the group promote (through shared guidelines) respect and sharing among one another?	–	–	Yes	Yes	–	Yes	Yes	Yes	–
Does the group allow for the participation of support persons?	–	–	–	Yes	–	–	Yes	No^a^	–
Are participants provided with the opportunity for socializing within the group?	–	–	–	Yes	Yes	–	Yes	Yes	–
Are discussions and activities conducted in a circle?	–	–	–	Yes	Yes	–	Yes	Yes	–
(e) Logistics									
What is the total number of group sessions?	4–6 ANC	4 ANC + 4 PNC	–	–	7 ANC	10 ANC	–	9 ANC	20
How frequently do the group sessions occur?	–	Monthly	–	–	–	–	–	–	Monthly
How long is each group session?	120 min	120 min	–	–	–	90–120 min	–	120 min	–
Do the leaders and/or participants regularly evaluate the group experience?	–	–	–	Yes	Yes	–	Yes	Yes	–

*CP*: Centering Pregnancy®; *CHW*: Community Health Worker; *HCP*: Healthcare Provider

^a^Support person participation was obligatory as study focuses on paternal satisfaction with the group model

**Table 2:** Summary of evidence from key informant interviews describing group antenatal care models in low- and middle-income countries

Key Informant Interview Identification Number	1	2	3	4	5	6	7	8	9	10
Panel I: Study or Program Characteristics										
Group ANC or Participatory Group or Hybrid	Group ANC	Group ANC	Group ANC	Group ANC	Hybrid	Hybrid	Hybrid	Group ANC	Group ANC	Group ANC
Country	Kenya	Suriname	MalawiTanzania	Rwanda	Kenya	Ghana	Uganda	Nigeria, Kenya	Mexico	Nepal
Setting	Urban Private Non-hospital	Urban--	Rural Malawi, Urban Tanzania--	Rural + urban--	Rural Public Non-hospital	Urban--	Rural + urban--	Rural + urban--	Rural + urban--	Rural--
Is the model informed by an existing group format?	No	CP	CP	CP	HBLSS	HBLSS	HBLSS	CPHBLSS	CP	CPParticipatory groups
Panel II: Model Attributes										
(a) Group										
How many women are in a group?	–	8–12 women	12 women	8–12 women	5 women	12 women	10–12 women	8–15 women	10 women	6–15 women
How are women grouped?	–	Gestational age	Gestational age	Gestational age	Gestational age	Gestational age	Gestational age	Gestational age	Gestational age	Gestational age
Is the composition of the group stable?	–	Yes	Yes	Yes	–	Yes	Yes	Yes	Yes	No
(b) Leaders										
How many leaders does the group have?	–	2	2	2	1	2	1	2	2	2
What cadre of care providers typically leads the group?	–	2 Midwives	1 Midwife1 CHW	1 Nurse/1 CHW	1 CHP	2 Midwives	1 Nurse or Midwife	2 Midwives	1 HCP/1 Nurse CHW	1 ANM1 CHW
Is the group leadership the same throughout all sessions?	Yes	Yes	Yes	Yes	–	Yes	Yes	Yes	Yes	Yes
Do group leaders use a facilitative leadership style?	–	Yes	Yes	Yes	–	–	–	Yes	Yes	Yes
(c) Content										
Does the model include a clinical assessment component?	Yes	Yes	Yes	Yes	Yes	Yes	Yes	Yes	Yes	Yes
Does the model include an education or skill-building component?	Yes	Yes	Yes	Yes	Yes	Yes	Yes	Yes	Yes	Yes
Does the model include a support group or counselling component?	Yes	Yes	Yes	Yes	Yes	Yes	Yes	Yes	Yes	Yes
Does the health assessment occur within the group space?	No	No	Yes	Yes	No	No	No	Yes	Yes	Yes
Do the women participate in self-care activities?	–	Yes	Yes	No	No	–	–	Yes	Yes	Yes
Are sessions guided by an overall plan or outline?	Yes	Yes	Yes	Yes	Yes	Yes	Yes	Yes	Yes	Yes
Do sessions follow the core content but allow flexibility to tailor the session to the group’s needs?	Yes	Yes	Yes	Yes	–	–	–	–	Yes	Yes
Does the model include a postnatal care component?	–	Yes (1 PNC)	Yes (1 PNC)	Yes (1 PNC)	–	–	–	–	Yes (1 PNC)	Yes (1 PNC)
Dynamics and Environment										
Does the group promote (through shared guidelines) respect and sharing among one another?	Yes	Yes	Yes	Yes	–	–	–	–	Yes	Yes
Does the group allow for the participation of support persons?	Yes	Yes	Yes	Yes	–	–	–	–	Yes	Yes
Are participants provided with the opportunity for socializing within the group?	Yes	Yes	Yes	Yes	–	–	–	–	Yes	
Are discussions and activities conducted in a circle?	Yes	Yes	Yes	Yes	–	–	–	–	Yes	Yes
(d) Logistics										
What is the total number of group sessions?	–	9–10 ANC + 1 PNC	4 ANC + 1 PNC	4 ANC + 1 PNC	–	7 ANC	5 ANC	–	7 ANC + 1 PNC	3 ANC + 1 PNC
How frequently do the group sessions occur?	–	–	–	–	–		–	–	–	Approx. every 2 months
How long is each group session?	–	–	–	90 min	–	60–90 min	–	–	120 min	–
Do the leaders and/or participants regularly evaluate the group experience?	–	Yes	Yes	Yes	–	–	–	–	Yes	Yes

Hybrid refers to a combination of elements of the HBLSS and CenteringPregnancy® models

*ANM*: Auxilliary Nurse Midwives; *CHW*: Community Health Workers; *CHP*: Community Health Provider; *CP*: CenteringPregnancy®; *HBLSS*: Home Based Life Saving Skills

### Evidence synthesis: Descriptions of the model

We developed a generic model of group ANC for LMIC settings that leaves room for further adaptation to meet local standards based on a synthesis of the evidence collected. The generic model is the generic model is described in Table 3. Below we summarize the supporting evidence for this model.

**Table 3:** A “generic” model of group antenatal care for low- and middle-income countries

Recruitment of women into group ANC takes place at the time of the first ANC visit, which follows the facility’s standard protocol. The “intake” visit follows the regular one-on-one format for ANC.
During this visit, the healthcare provider confirms pregnancy, performs initial lab tests and a physical exam, and screens for high-risk conditions. Pregnant women are then invited to join a group of 8-12 women with similar due dates to receive antenatal care in a group setting. If a woman chooses group ANC, she will be given the schedule for all her group care visits through the end of her pregnancy. She will receive care with the same group of women each time, and is expected to attend each of her group’s sessions, to help create a stable cohort.
The number of group ANC sessions may be tailored to match the number of visits recommended by global and local guidelines.
During the first group session, the women decide as a group whether they want support persons (for example, husband, mother, mother-in-law or sister) to participate in the sessions. Each session is facilitated by two group leaders, one of whom is a healthcare provider who can provide clinical care. Each session lasts 90 to 120 minutes, and has three parts: physical assessment, learning and education, and peer support.
Each group ANC session begins with self-assessments by the pregnant women and a physical assessment by a healthcare provider. During the first 30 minutes, one of the group leaders (for example, a nurse, medical assistant, or community health worker) helps the women take their own basic health measurements, such as blood pressure and weight, and reflect on some predetermined aspect of their physical and emotional wellbeing. Women may also be asked to think about or fill out a worksheet on a topic, which is used to inform the group discussion later. During this time, the other group leader—who must be a healthcare provider (for example, doctor, nurse, or midwife)— conducts the physical assessment for each woman, one at a time. This basic physical exam follows the ANC clinical guidelines recommended by the World Health Organization and national authorities. It takes place in a private area (like a corner) of the group space, and care is taken to ensure that each woman’s auditory and visual privacy and confidentiality are protected (for example, through the use of music or a screen or curtain).
After each of the assessments is completed, the women come together for the remainder of the session for group activities and discussion. During the discussions, the women and the providers sit together in a circle and take turns sharing, making sure that everyone has a chance to speak without interruption. The group leaders use a facilitative leadership style to promote the discussion. Using this style, they do not lecture to the women like in a classroom, but instead facilitate a discussion of the topics planned for the session and contribute to the discussion themselves along with the women. This part of the session is an opportunity for women to talk about how they are feeling, ask questions and share information with each other and the providers, build supportive relationships, and learn about pregnancy and birth. There is also time within each group session for informal socializing.
Each group session has a plan that includes specific content for clinical care and client education. Nevertheless, the session plan is flexible enough to make sure that the discussion is always relevant to the women and addresses their specific needs. Throughout the course of a group’s ANC sessions, there are opportunities for the women to provide feedback about their experiences in the group. This information can be used by the group leaders to evaluate and improve the program. There are also opportunities after each session for the group leaders to discuss how the group went and to talk about any areas for improvement in the group leadership or any clinical issues that need follow up.

Group sizes ranged from 5 to 20 women, but most (13/19) reported group size of 8–12 women [26, 30, 31, 33, 35–42]. Most published studies (7/9) [26, 27, 30–34] and ongoing projects (9/10) [36–44] described groups composed of women of similar gestational age; only one study reported grouping women based on availability and willingness to participate [29]. Key informants noted that consistency of group members ensures that topics discussed in each session are relevant to all participants, promotes trust and cohesion among the women, and a sense of belonging and commitment to the group. Only one study reported not having a stable cohort of pregnant women following through all the ANC sessions [42].

Of the studies reporting on group leadership (12/19) all maintained the same group leaders throughout the course of the ANC sessions [29, 32, 33, 36–42, 44, 45]. Most studies (10/19) had two group leaders, at least one of whom is a certified healthcare provider [26, 27, 36–38, 40–42, 44]; a few (3/19) had only one group leader [31, 39, 43]. The use of a facilitative leadership style was described by all studies reporting on this aspect (11/19) [29, 31–34, 36–38, 40–42], and key informants viewed this as key in ensuring that the group remains woman-centered and interactive.

A clinical assessment piece, an education or skill-building activities piece, and a support group or counseling piece were included in all group ANC models (18/19) [26–33, 36–45]; only the participatory group model described in one study [34] did not have a clinical assessment component. Of the models that included a clinical component, the majority conducted the women’s individual physical assessments within the group space, in a designated, private area [28–30, 32, 33, 37, 38, 40–42]. One published study on group ANC and half (5/10) of the key informants reported that the health assessment did not occur in the group space [31, 36, 39, 43–45]. Most (16/19) models described involved women in self-care activities (ex. measuring blood pressure and weight) [28, 29, 31–33, 36, 37, 40–42].

Sixteen out of 19 sources analyzed reported that group sessions were guided by an educational plan [26, 29–33, 36–45]. All key informants (*n* = 10) reported that their models followed an overall content plan, but allowed flexibility to tailor the session to the group’s needs [36–45]. Inclusion of postnatal care (PNC) sessions was variable: only 7/19 models reported including at least one PNC session [27, 33, 36–38, 41, 42].

Mechanisms to encourage peer support and relationship building within the group were described in most models. A majority reported use of mechanisms to support group equality: democratic conduct that reflects respect for all participants (11/19) [28, 29, 31–33, 36–38, 41, 42, 45], designated time for socializing within the group (9/11) [29, 30, 32, 33, 36–38, 41, 45], and group seating in a circle (10/19) [29, 30, 32, 33, 36–38, 41, 42, 45]. While not all sources reported on this attribute, some group care models (8/19) also accommodated support persons (such as husbands, mothers, or mothers-in-law) if women desired their involvement [29, 32, 36–38, 41, 42, 45]. A few models enabled women to discuss and decide during the first group session whether, and how, to include support persons.

Although the specific number of sessions varied among the group ANC models we reviewed, all the models that reported on the number of visits included at least four ANC visits (13/19) [26, 27, 30, 31, 33, 36–39, 41, 42, 44]. This aligns with the previous WHO guidelines for a minimum of four ANC visits [46]; more recent revisions to the guidance offered by WHO on ANC recommends eight “contacts,” but these are yet to be widely adopted by countries [47]. Key informants noted that the number of sessions was selected to meet the minimum requirements mandated by national standards in their respective study or program settings. Session duration varied across the models that reported on this attribute, ranging from a minimum of 60 min to a maximum of 120 min (7/19) [26, 27, 31, 33, 38, 41, 44]. Of those records (9/19) that reported on monitoring and evaluation, all indicated that mechanisms for ongoing evaluation were built into their group care model [29, 30, 32, 33, 36–38, 41, 42]. On-going evaluation of the group process and monitoring of the outcomes of interest (defined by the organization, administrators, and healthcare professionals providing the group care) was perceived as essential to ensuring that the group model effectively delivers high-quality care and positive experiences for women.

## Discussion

The World Health Organization recognizes group ANC provided by qualified health-care professionals as a health system intervention that provides an alternative to individual ANC and has the potential to improve utilization and quality of care for pregnant women [47]. Introducing group ANC in LMICs can offer an opportunity to examine and improve delivery, performance and utilization of services for pregnant women, especially in settings where coverage of comprehensive care is low and the quality of care is poor [48–50]. In alignment with the World Health Organization’s framework for quality of care [51], group ANC models put women at the center of service provision and aim to improve women’s access, engagement, and satisfaction with care.

Through a systematic review of published literature and expert consultations, we synthesized evidence from 19 models of group care for pregnant women and identified attributes that appeared consistently across all group care models and could be considered fundamental to the effective delivery of group ANC. These include, for instance, providing a physical assessment, using facilitated discussion to foster learning and peer support, and including women in self-care activities. We also identified attributes that required flexibility: features that need to be tailored to the context in which the model is implemented, for instance, number of sessions or the session content. This combination of standard and flexible components is key when planning for implementation across LMIC settings. This “generic” model synthesized from all available sources of data on group ANC in LMICs ensures conformity with the best available evidence while maintaining pliability to accommodate contextual differences.

Several components of the “generic” model aim to empower and support women. For example, engaging in discussion and shared care with other women of similar gestational age helps to normalize the experience of pregnancy, and gives women a voice for knowledge sharing and a sense of community for support. The group format also fosters self-efficacy and social support for pregnant woman by creating a forum for participants to build skills and confidence, share experiences and resources, and socialize with one another. Likewise, the facilitative leadership style ensures that the group remains woman-centered and interactive, allowing participants to learn from each other and address concerns relevant to the group. Having a voice in their care is a key component of patient activation and empowerment, ensuring that women are active participants and not passive recipients of care and information [34, 52]. Literature on shared medical appointments and group therapy suggests that a range of 7 to 12 individuals per group is most effective to support group processes, allowing participants a chance to speak without making them feel uncomfortable or exposed [53, 54]. This is supplemented with mechanisms to support group equality: democratic conduct that reflects respect for all participants, designated time for socializing within the group, and group seating in a circle. Collectively, these features help overcome power differences between patients and providers that can act as a barrier to patient engagement in maternal health care [34]. Finally, consistency of both leadership and group membership promotes bonding and trust between the women and the care providers and allows for continuity of clinical care, an important aspect of quality [34].

Providing ANC in a group setting offers increased convenience for women and providers, and can make care delivery more efficient. Long wait-times for care have been cited as a barrier to utilization of maternal and newborn health services in LMICs [55, 56]. Scheduling group sessions in advance, a necessary feature in group ANC, could help overcome this barrier. Additionally, in contrast to a one-on-one model, delivering care in a group setting allows sufficient time for all women to receive recommended clinical care and offers opportunities for counseling, enabling women to benefit from the expertise and support of their healthcare providers and peers. Such engagement between providers and women allows for both efficient and comprehensive care delivery, which in turn may improve the provision, experience, and utilization of care and offer opportunities to ensure continuity of care throughout the reproductive, maternal, newborn and child health continuum.

The attributes of the composite generic model we codified from this comprehensive evidence synthesis are well aligned with the “essential elements” of CenteringPregnancy®. Thus, the findings of our systematic scoping exercise and synthesis of common model elements in use in LMICs supports the findings by Patil et al. that the essential elements of the Centering® model are feasible and appropriate for use in LMICs, with context-specific adaptation to meet local practice guidelines [35].

### Limitations and strengths

Our systematic scoping review and resulting composite model have several limitations, primarily owing to the lack of published evidence. Although our literature search strategy was carefully developed to be comprehensive, it may not have captured all relevant published literature, as the keywords and search terms are not well defined or established at this stage, and we limited our search to articles in English. Additionally, the models described in the published literature were not consistently detailed. Similarly, our key informants were selected from a small network of researchers and program implementers, and therefore do not necessarily capture the perspectives of all those currently working with group ANC in LMICs.

Despite these limitations, our study is a relevant addition to the nascent evidence base of resources on adapting group ANC for LMICs. To our knowledge, this is the first systematic review and evidence synthesis related to group ANC in LMICs. By both reviewing published literature and consulting with experts, we systematically analyzed and synthesized multiple models for providing group ANC in LMIC settings. We identified those aspects that are fundamental for a group ANC model to provide high-quality care and should be standard, as well as those that require flexibility to ensure contextual relevance. Additionally, by supplementing the data gathered from published evidence with that gathered through expert consultation, the resulting composite “generic” model of ANC builds on real-time lessons and experiences from the field. It is hoped that this systematic scoping review will be useful to inform future research and programs aiming to introduce and implement group ANC in LMICs.

## Conclusion

Through a systematic scoping review and synthesis of evidence on group care models for pregnant women, we have compiled an evidence-informed “generic” model of group ANC that may be used in efforts to improve the provision and experiences of care for pregnant women in LMICs. Effectiveness research to test the group ANC model’s effectiveness for improving quality across a range of dimensions, including utilization of services, maternal and newborn health outcomes, and experiences for pregnant women in LMICs, is needed. In addition, future collaborative research could bring together researchers and programmers who have introduced group ANC in LMICs, along with the women who have experienced it, to share lessons and present best practices for adapting an evidence-based intervention designed in a high-income setting for specific use in LMICs. Such research would be useful to inform ongoing efforts to improve the provision and experiences of health care services globally.

## Additional files

Additional file 1: Database search strategy. (DOCX 21 kb)

Additional file 2: Semi-structured interview guide for key informant interviews. (DOCX 14 kb)

## Abbreviations

ANC: Antenatal care
HBLSS: Home Based Life Saving Skills
HCP: Health care provider
LMIC: Low- or middle-income country
PNC: Postnatal care
