# Supplementary material for: Group antenatal care models in low- and middle-income countries: a systematic evidence synthesis
Source: Reprod Health. 2018 Mar 5;15:38. doi: 10.1186/s12978-018-0476-9 (PMC5836451; doi:10.1186/s12978-018-0476-9)
Supplement: Supplementary file 1 — Database search strategy. (DOCX 21 kb) [file 12978_2018_476_MOESM1_ESM.docx]

**Additional file 1: Database search strategy and terms**

The search strategy and terms used for each database are below. In addition to these search terms, a low- and middle- income country hedge was applied to each search.

**MEDLINE**

*including Epub Ahead of Print, In-Process & Other Non-Indexed Citations, Ovid MEDLINE(R) Daily and Ovid MEDLINE(R)*

*((centeringpregnancy OR centering pregnancy).ab,ti*

*OR*

*((exp Prenatal Care/ OR ((prenatal OR pre natal OR antenatal OR ante natal OR perinatal OR peri natal) adj (care OR control OR education OR intervention)).ab,ti) AND (Group Processes/ OR Peer Group/ OR (group? adj (based OR meeting? OR session? OR setting? OR thinking OR discussion? OR dynamic? OR support OR intervention?)).ab,ti OR ((wom?n$ OR support) adj group?).ab,ti))*

*OR*

*(((group OR peer centered OR participatory) adj3 (antenatal OR ante natal OR prenatal OR pre natal OR perinatal OR peri natal) adj (care OR intervention?)).ab,ti))*

**Embase (Elsevier)**

*((centeringpregnancy:ab,ti OR 'centering pregnancy':ab,ti)*

*OR*

*(('prenatal care'/exp OR ((prenatal OR 'pre natal' OR antenatal OR 'ante natal' OR perinatal OR 'peri natal') NEXT/1 (care OR control)):ab,ti) AND ('group process'/de OR 'peer group'/de OR ((group OR groups) NEXT/1 (based OR meeting* OR session* OR setting? OR thinking OR discussion* OR dynamic* OR support OR intervention*)):ab,ti OR ((women* OR woman* OR support) NEXT/1 group*):ab,ti))*

*OR*

*(((group OR 'peer centered' OR participatory) NEAR/3 (antenatal OR 'ante natal' OR prenatal OR 'pre natal' OR 'perinatal' OR 'peri natal' OR 'postnatal' OR 'post natal')):ti,ab AND ((antenatal OR 'ante natal' OR prenatal OR 'pre natal' OR 'perinatal' OR 'peri natal' OR 'postnatal' OR 'post natal') NEXT/1 (care OR intervention*)):ab,ti))*

**Web of Science**

*TS=("centeringpregnancy" OR "centering pregnancy")*

*OR*

*(TS=(("prenatal" OR "pre natal" OR "antenatal" OR "ante natal" OR "perinatal" OR "peri natal") NEAR/1 ("care" OR "control"))*

*AND*

*TS=(("group" OR "groups") NEAR/1 ("based" OR "meeting*" OR "session*" OR "setting*" OR "thinking" OR "discussion*" OR "dynamic*" OR "support" OR "intervention*" OR "women*" OR "woman*" OR "support")) )*

*OR*

*(TS=(("group" OR "peer centered" OR "participatory") NEAR/3 ("antenatal" OR "ante natal" OR "prenatal" OR "pre natal" OR "perinatal" OR "peri natal" OR "postnatal" OR "post natal"))*

*AND*

*TS=(("antenatal" OR "ante natal" OR "prenatal" OR "pre natal" OR "perinatal" OR "peri natal" OR "postnatal" OR "post natal") NEAR/1 ("care" OR "intervention*"))*

**CINAL (EBSCO)**

*((TI (centeringpregnancy OR "centering pregnancy") OR AB (centeringpregnancy OR "centering pregnancy"))*

*OR*

*(MH "Prenatal Care" OR TI ((prenatal OR "pre natal" OR antenatal OR "ante natal" OR perinatal OR "peri natal") W1 (care OR control OR education OR intervention)) OR AB ((prenatal OR "pre natal" OR antenatal OR "ante natal" OR perinatal OR "peri natal") W1 (care OR control OR education OR intervention))) AND (MH ("Group Processes" OR "Peer Group") OR TI (group* W1 (based OR meeting* OR session* OR setting* OR thinking OR discussion* OR dynamic* OR support OR intervention*)) OR AB (group* W1 (based OR meeting* OR session* OR setting* OR thinking OR discussion* OR dynamic* OR support OR intervention*)) OR TI ((wom?n* OR support) W1 group*) OR AB ((wom?n* OR support) W1 group*))*

*OR*

*TI ((group OR "peer centered" OR participatory) W3 (antenatal OR "ante natal" OR prenatal OR "pre natal" OR perinatal OR "peri natal") W1 (care OR intervention*)) OR AB ((group OR "peer centered" OR participatory) W3 (antenatal OR "ante natal" OR prenatal OR "pre natal" OR perinatal OR "peri natal") W1 (care OR intervention*)))*

**WHO Global Health Library (excluding MEDLINE)**

*centeringpregnancy OR "centering pregnancy" OR "group prenatal care" OR "group pre natal care" OR "group antenatal care" OR "group ante natal care" OR (("support group" OR "support groups" OR "group meeting" OR "group meetings" OR "group session" OR "group sessions" OR "group setting" OR "group intervention" OR "group interventions" OR "women's group" OR "woman's group") AND ("antenatal care" OR "ante natal care" OR "prenatal care" OR "pre natal care"))*
