# Supplementary material for: Group antenatal care models in low- and middle-income countries: a systematic evidence synthesis
Source: Reprod Health. 2018 Mar 5;15:38. doi: 10.1186/s12978-018-0476-9 (PMC5836451; doi:10.1186/s12978-018-0476-9)
Supplement: Supplementary file 2 — Semi-structured interview guide for key informant interviews. (DOCX 14 kb) [file 12978_2018_476_MOESM2_ESM.docx]

**Additional file 2: Semi-structured interview guide for key informant interviews**

*Please describe your project to us.*

1. What is the purpose of your project?
   1. Is it primarily research?
   2. Is it a care delivery program?
   3. Are you testing or implementing?
   4. If this project is strictly research, what are the study objectives?
2. Where is your project located (country, urban/rural)?
3. Is the project setting a clinical facility or is it community-based?
4. How long has your project been active?
5. How is your project funded?

*Please describe the group ANC model your project is using.*

1. What are the elements of your model?
   1. What is the average group size?
   2. What method is used to group the participants (gestational age, language, etc.)?
   3. Who is the facilitator (doctor, midwife, nurse, etc.)?
   4. What is the role of the healthcare provider?
2. Are you using the CenteringPregnancy® model?
   1. YES: Are you using all 13 essential elements?
   2. YES: Are you modifying the model at all and if so, why?
   3. NO: How did you develop your model?
3. Have you piloted the model?
4. Have you implemented the model?
   1. YES: What have been some of the challenges with implementation?
5. Are you including any monitoring and evaluation or process improvement in your model?
6. Overall, what is working well?
7. Overall, are there any elements or aspects of the model you are using that are challenging or not particularly effective?
   1. What changes – if any – would you make to the model if you were to do it again?
8. What have been some of the primary and secondary outcomes of the project?
